# Cross-Linked Thiolated Hydroxypropil-β-Cyclodextrin for Pulmonary Drug Delivery

**DOI:** 10.3390/ijms25179394

**Published:** 2024-08-29

**Authors:** Luca Cerri, Chiara Migone, Lucia Vizzoni, Brunella Grassiri, Angela Fabiano, Anna Maria Piras, Ylenia Zambito

**Affiliations:** 1 Department of Pharmacy, University of Pisa, Via Bonanno 33, 56126 Pisa, Italy; luca.cerri@unipi.it (L.C.); chiara.migone@unipi.it (C.M.); l.vizzoni@student.unisi.it (L.V.); brunella.grassiri@phd.unipi.it (B.G.); angela.fabiano@unipi.it (A.F.); ylenia.zambito@unipi.it (Y.Z.); 2 Department of Life Sciences, University of Siena, 53100 Siena, Italy; 3 Center for Instrument Sharing of the University of Pisa (CISUP), University of Pisa, 56126 Pisa, Italy; 4 Research Centre for Nutraceutical and Healthy Foods “NUTRAFOOD”, University of Pisa, 56126 Pisa, Italy

**Keywords:** cyclodextrins, nanocarrier, drug delivery, mucoadhesion, natural extract, lung delivery

## Abstract

Inhalable formulations with cyclodextrins (CDs) as solubility and absorption enhancers show promise for pulmonary delivery. Thiolated hydroxypropyl-β-cyclodextrin (HP-β-CD-SH) has mucoadhesive properties, enhancing drug absorption. Moreover, it has self-aggregation capability, which could further improve absorption and drug stability, as well as reduce irritation. This study aims to stabilize CD nanoaggregates using bifunctional cross-linkers and evaluate their benefits for lung drug delivery compared to pristine HP-β-CD-SH. Methods: The effectiveness of cross-linked HP-β-CD-SH nanoparticles (HP-β-CD-SH-NP) was compared to transient nanoaggregates in enhancing the activity of dexamethasone (DMS) and olive leaf extracts (OLE). DMS, a poorly soluble drug commonly used in lung treatments, and OLE, known for its antioxidant properties, were chosen. Drug-loaded HP-β-CD-SH-NP were prepared and nebulized onto a lung epithelial Air–Liquid Interface (ALI) model, assessing drug permeation and activity. Results: HP-β-CD-SH with 25% thiolation was synthesized via microwave reaction, forming 150 nm nanoaggregates and stabilized 400 nm HP-β-CD-SH-NP. All carriers showed good complexing ability with DMS and OLE and were biocompatible in the lung ALI model. HP-β-CD-SH promoted DMS absorption, while stabilized HP-β-CD-SH-NP protected against oxidative stress. Conclusion: HP-β-CD-SH is promising for lung delivery, especially as stabilized nanoaggregates, offering versatile administration for labile molecules like natural extracts.

## 1. Introduction

Inhalation is a widely employed method of drug administration, serving as a pivotal route for various medical applications. It proves instrumental for the targeted delivery of local drugs, such as those for bronchial diseases or for chemotherapy. Simultaneously, it plays a crucial role in administering systemic drugs, including insulin therapy and gene therapy [1]. This versatile approach ensures the effective and efficient distribution of medications, offering tailored solutions for a spectrum of medical needs [2]. Respiratory diseases currently represent an enormous and growing health and economic burden across Europe, with about 350,000 deaths and 4.2 million hospitalizations per year in 2020 [3]. Despite growing medical needs in respiratory medicine, there has been limited introduction of new effective drugs in the past 40 years. Respiratory medicine lags behind other fields like cardiovascular, metabolic, or neurological medicine, with fewer innovative therapies and a higher rate of failures [4]. Positive perspectives are arising for the development of aerosolized formulations containing cyclodextrins (CDs) as solubility and absorption enhancers for pulmonary delivery [5,6].

CDs are cyclic oligosaccharides consisting of α-D-glucopyranoside units and linked in a cyclic form by α-(1-4)-glycosidic bonds. CDs are employed in pharmaceutical formulations for delivering poorly soluble drugs or forming complexes with natural extracts. The formation of drug (guest)–CD (host) complexes with poorly soluble molecules in aqueous solution induces chemical–physical changes in the guest molecule. This complexation results in increased stability by protecting active principles against oxidative and thermal degradation, hydrolysis, and by masking unpleasant odors and tastes [6,7]. 

To the best of our knowledge, Miyajima and his colleagues were the first to suggest that αCD and γCD self-associate to form aggregates in aqueous solutions [8]. CDs were found to form transient clusters that dissociate during the manipulation of the CD dispersion in water and, therefore, are difficult to detect by spectroscopic techniques. With the advent of new technologies for nanosystem studies, including dynamic light scattering, CD self-aggregates have received increasing attention from researchers. It has been demonstrated that with the formation of CD nanoaggregates, biopharmaceutical benefits are obtained—for example, better penetration of CD aggregates through mucus [9], or better promotion of ocular bioavailability of topically administered drugs compared to non-aggregated CDs [10]. In ocular applications, CDs contribute to forming water-soluble complexes, improving drug absorption, ensuring aqueous stability, and minimizing irritation [11]. Another process, namely crystallization, can occur simultaneously or as a consequence of aggregation. This can lead to a decrease of solubility and worse pharmacokinetic properties of the guest API [11].

Recently, a derivative of hydroxypropyl-β-cyclodextrin (HP-β-CD), thiolated hydroxypropyl-β-cyclodextrin (HP-β-CD-SH), has been synthesized, exhibiting notable mucoadhesive properties, forming disulfide bonds with cysteine-rich subdomains of mucus glycoproteins, thus further promoting drug absorption compared to the parent CD [12]. Additionally, it has recently been reported that nanoaggregates are formed at very low concentrations when thiolated cyclodextrins are applied [13]. 

Hence, the objective of this work has been to stabilize CD nanoaggregates with bifunctional cross-linkers and evaluate their advantages over not-stabilized CD aggregates for drug delivery to the lung. The ability of cross-linked HP-β-CD-SH nanoparticles to promote the activity of two different active ingredients, namely dexamethasone (DMS) and olive leaf extracts (OLE), was compared to that of transient CD nanoaggregates. DMS was chosen as a model drug due to its poor solubility in water and its widespread use in lung administration, while OLE was chosen because of the usefulness of natural polyphenols for the treatment of inflammatory lung diseases [14] and for its antioxidant activity on lung cells [15,16]. So, drug-loaded cross-linked HP-β-CD-SH nanoparticles have been prepared and nebulized onto a lung epithelial Air–Liquid Interface (ALI) cellular model, widely accepted as a physiologically relevant model for respiratory research [17]. The DMS permeation and the antioxidant effect of OLE in comparison to formulations based on non-cross-linked HP-β-CD-SH or HP-β-CD have been evaluated.

## 2. Results

### 2.1. Synthesis and Characterization of HP-β-CD-SH-NP

**Figure 1:**
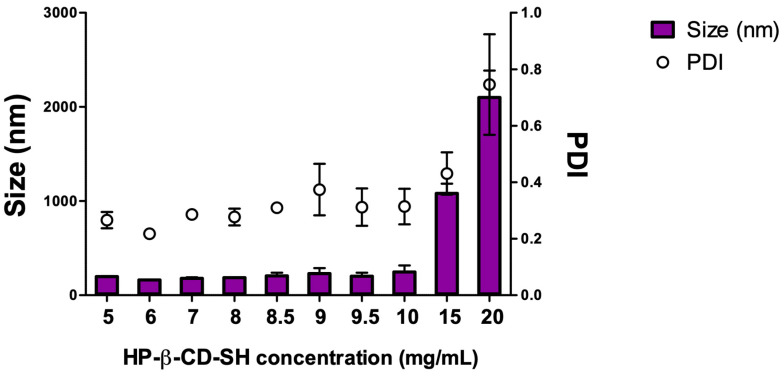
Average diameter (bar) and polydispersity index (PDI; empty circles) of HP-β-CD-SH dispersions in DPBS, at increasing concentrations. Means ± SD (*n* = 3).

HP-β-CD-SH was obtained starting from HP-β-CD via microwave reaction as reported by Grassiri et al. [12] (Supporting Information Figure S1). ^1^H NMR analysis (Supporting Information Figure S2) has shown a 25% thiolation degree of HP-β-CD-SH, not significantly different from that obtained previously [12]. HP-β-CD-SH dispersions were tested for size at concentrations ranging from 5 to 20 mg/mL HP-β-CD-SH in DPBS, to verify the spontaneous formation of nanometer-sized aggregates, as previously observed with other commercial and thiolated cyclodextrins [13,18]. Figure 1 shows the dimensions and PDIs of the dispersions tested. The formation of aggregates having nanometric dimensions and a low PDI is observed at concentrations between 5 and 10 mg/mL, while at higher concentrations, a remarkable increase in size and PDI is seen. Among the concentrations of HP-β-CD-SH leading to the formation of nanoaggregates, the highest concentration, i.e., 10 mg/mL, was chosen, as this makes it possible to solubilize a greater quantity of drug. At the concentration chosen, cyclodextrins form unstable nanoaggregates that form and break down continuously, as already reported for HP-β-CD [19].

**Figure 2:**
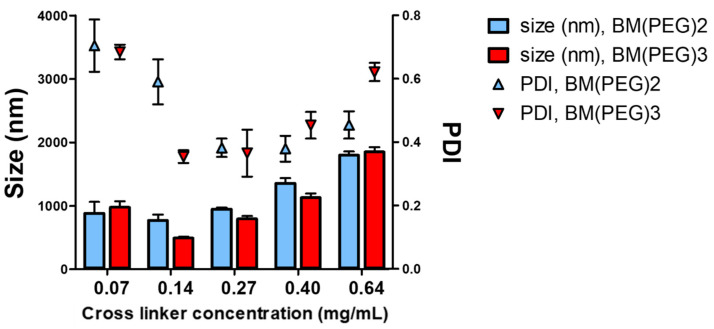
Influence of the reaction of two different types of cross-linker, BM(PEG)2 or BM(PEG)3, at different concentrations with HP-β-CD-SH on HP-β-CD-SH-NP size (bars) and PDI (points). Means ± SD (*n* = 3).

In order to obtain stable nanoparticles (HP-β-CD-SH-NP), two different cross-linkers capable of forming thioether bonds with the thiol groups present on HP-β-CD-SH were chosen. In particular, the cross-linkers tested, i.e., BM(PEG)2 and BM(PEG)3, are homobifunctional with a spacer arm made up of hydrophilic polyethylene glycol groups, which give the molecule good water solubility. The maleimide moiety, present in both cross-linkers, is temporarily stable in aqueous solutions free of reactive sulfhydryl targets, but hydrolysis to non-reactive maleic acid may occur during storage, especially at pH > 8 (Supporting Information Figure S3). For this reason, it is advisable to use them promptly [20]. The interaction between the sulfhydryl and the maleimide moieties, belonging to the HP-β-CD-SH and the cross-linker, respectively, results in the formation of a stable thioether bond. This bond exhibits resistance to cleavage by reducing agents. Notably, the maleimide reaction with sulfhydryls is highly specific in the 6.5–7.5 pH range [21]. In the tuning of the cross-linking conditions, different quantities of the two different cross-linkers were used following dimensional analysis to choose the best conditions for the formation of nanoaggregates. In particular, the final concentrations 0.07, 0.14, 0.27, 0.40, and 0.64 mg/mL, which fell perfectly within the range recommended by the manufacturer, were tested for both BM(PEG)2 and BM(PEG)3 in the presence of 10 mg/mL HP-β-CD-SH solution. Figure 2 shows the dimensions and PDIs of the aggregates that formed under the conditions described. The data reported in Figure 2 show that the use of the BM(PEG)3 cross-linker, at all the concentrations tested, made it possible to obtain nanoparticles that were smaller or at least not significantly different in size from those obtained with the BM(PEG)2 cross-linker. It is also observed that at the BM(PEG)3 concentration of 0.14 mg/mL, nanoparticles of smaller size and with one of the lowest PDI were obtained; therefore, these conditions were chosen for the preparation of HP-β-CD-SH-NP in all subsequent experiments. The dispersion thus obtained was centrifuged, after which the formation of a transparent precipitate with a gelatinous consistency was observed. This was further evidence of the formation of nanoaggregates [22]. Furthermore, compared to the reagents in solution present at time zero, the dry weight of the cross-linked product was determined, and the reaction yield was 21.9%. 

### 2.2. Determination of the Association Constant for Cyclodextrins/DMS and Cyclodextrins/OLE-GS

Cyclodextrins form inclusion complexes with lipophilic molecules, with a consequent increase in the apparent solubility of the latter. A dynamic balance is therefore established between complexed and free forms. The association constants (K_a_) of the cyclodextrin/DMS and cyclodextrin/OLE-GS complexes were determined with the Benesi–Hildebrand method [23] in association with UV-vis spectrometry exploiting the variation in the absorption of solutions having the same content of DMS or OLE-GS and variable concentrations of cyclodextrins. K_a_ was calculated using the Benesi-Hildebrand equation [24]:

1/(A − A0) = 1/(A′ − A0) × 1/[CD] + 1/(A′ − A0)(1)

where [CD] represents the concentration of cyclodextrin, A and A0 are the absorbances, in the presence and absence of CD, respectively, and A′ is the absorbance when all DMS molecules are complexed with CD. For each cyclodextrin tested, a straight line was obtained based on the equation above. The K_a_ was calculated by dividing the intercept by the slope of the relevant straight line. Table 1 shows the K_a_ obtained. Despite OLE-GS being a mixture of different compounds, the K_a_ was calculated based on a coherent UV physical variation. However, it must be taken into account that this does not represent a single molecule complexing, but it is an indication of the affinity of the OLE-GS mixture for the cyclodextrins under study.

The value of K_a_ relating to the complexation of DMS with HP-β-CD, although comparable, is not identical to that reported in other papers [25]. However, it must be kept in mind that K_a_ is greatly affected by the difference between the various sources of the products used and the method used to determine it. For this reason, in the subsequent discussion, only the relative differences between the various complexes were taken into account, rather than the absolute values of K_a_.

As can be seen, HP-β-CD shows a lower ability than either HP-β-CD-SH or HP-β-CD-SH-NP to form complexes with either DMS or OLE-GS. On the other hand, HP-β-CD-SH shows the best ability to complex either DMS or OLE-GS, even compared to HP-β-CD-SH-NP. It has been reported in the literature that native cyclodextrins have a lower solubility in water than their hydroxypropyl derivatives. This phenomenon is thought to influence the ability of cyclodextrins to form complexes with drugs [26]. Indeed, the random replacement of the hydroxyls with hydroxypropyl groups transforms the native crystalline cyclodextrins into amorphous mixtures of different isomers. Probably, the further replacement of the hydroxyls with -SH groups leads to the formation of an even more soluble product, and therefore one more capable of complexing. On the other hand, the cross-linking of HP-β-CD-SH with the formation of HP-β-CD-SH-NP makes the molecules structurally more rigid and less capable of interacting with the active ingredients under study.

### 2.3. Cytotoxicity Studies

**Figure 3:**
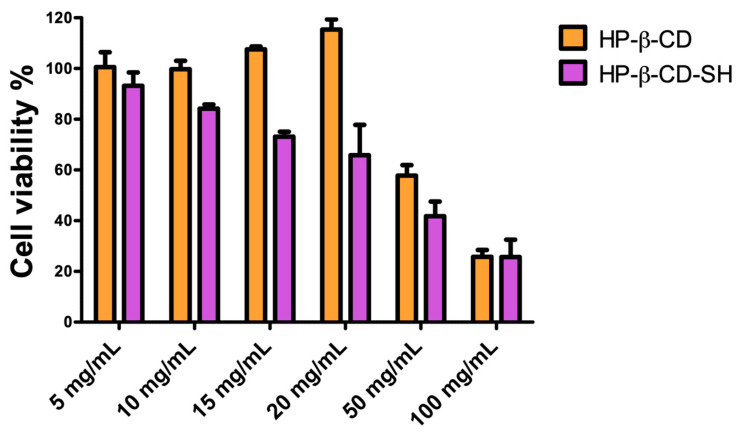
NCI-H441 viability after 4 h treatment with HP-β-CD or HP-β-CD-SH in culture medium. Data are expressed as % viable cells compared to control (untreated cells). Means ± SD, *n* = 6.

The cytotoxicity of HP-β-CD and HP-β-CD-SH tested on NCI-H441 cells in a 5–100 mg/mL concentration range was evaluated (Figure 3). The concentration of 10 mg/mL, chosen for technological reasons, is ideal for subsequent nebulization studies on monolayers, as at this concentration the two cyclodextrins does not show any toxicity, although HP-β-CD-SH has been shown to be more toxic than HP-β-CD. These data are in agreement with those already obtained with BALB/3T3 [12], where HP-β-CD-SH was found to be more toxic than HP-β-CD, but safe to use.

**Figure 4:**
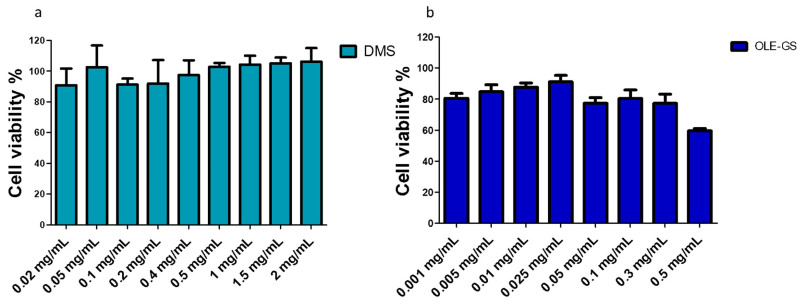
NCI-H441 viability after 4 h treatment with: (**a**) DMS and (**b**) OLE-GS in culture medium. Data are expressed as % viable cells compared to control (untreated cells). Means ± SD, *n* = 6.

The cytotoxicity of DMS and OLE-GS on the NCI-H441 cell line was also assessed. Figure 4 shows the cytotoxicity study on cells treated with increasing concentrations of DMS (Figure 4a) or OLE-GS (Figure 4b). The data in Figure 4a show that in the 0.02–2 mg/mL concentration range, DMS is not cytotoxic. For this reason, in subsequent studies, DMS was tested at 0.25 mg/mL, i.e., the concentration that, on the basis of K_a_, remained dissolved with all types of the cyclodextrins under study. In Figure 4b, the OLE-GS tested at a concentration in the 0.001–0.5 mg/mL range shows cell viability values close to 80% up to a concentration of 0.3 mg/mL, which then drops below 70% viability at a concentration of 0.5 mg/mL.

**Figure 5:**
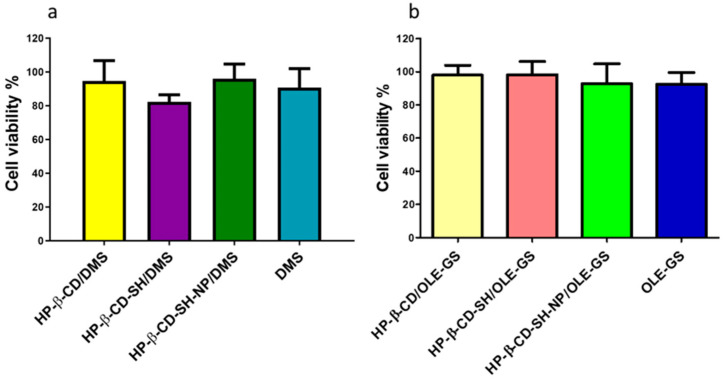
NCI-H441 viability after 4 h treatment with: (**a**) DMS (0.25 mg/mL), HP-β-CD/DMS, HP-β-CD-SH/DMS, HP-β-CD-SH-NP/DMS and (**b**) OLE-GS (0.3 mg/mL), HP-β-CD/OLE-GS, HP-β-CD-SH/OLE-GS, HP-β-CD-SH-NP/OLE-GS. Cyclodextrins concentration was always 10 mg/mL. Data are expressed as % viable cells compared to control (untreated cells). Means ± SD, *n* = 6.

Moving to the application on the ALI lung model of the selected formulations, containing both the cyclodextrin derivatives and the drugs, the verification of their cytocompatibility on the differentiated ALI in vitro model was performed (Figure 5). No significant cytotoxicity was detected, displaying cell viability values higher than 80% for either DMS (Figure 5a) or OLE-GS (Figure 5b) based formulations.

### 2.4. Dimensional Analysis and Zeta Potential of Drug-Loaded Formulations

HP-β-CD, HP-β-CD-SH and HP-β-CD-SH-NP dispersions complexed or not with DMS or OLE-GS were characterized in terms of size distribution and ζ potential (Table 2). Under the studied conditions, nano-size agglomerates are formed, in the rank order HP-β-CD < HP-β-CD-SH < HP-β-CD-SH-NP. The same trend is maintained for the complexes with DMS and those with OLE-GS, showing higher dimensions compared to the non-medicated samples, and a relatively smaller increase for the formulations loaded with OLE-GS compared to those with DMS. The PDIs of the drug-loaded NP dispersions were all lower than 0.5, and, in all cases, a lower PDI for the HP-β-CD-SH-based dispersions was obtained. It is not surprising that the PDI of these systems is not as low as desirable; it is in fact reported that they are composed of single cyclodextrin molecules in equilibrium with small clusters, in turn in equilibrium with larger ones [27] The ζ potential is significantly negative in all cases, but it is increasingly less negative in the case of drug-loaded and not-drug-loaded HP-β-CD-SH-NP. Moreover, the negative charge indicates that the hydroxyl groups of the CD, as well as the thiol ones, are facing the external surface, i.e., towards the aqueous environment, making the nanosystems hydrophilic. Since some thiol groups are involved in the formation of cross-links with PEG-based bridges in the stabilized HP-β-CD-SH-NP, the zeta potential value is affected by the reduced availability of thiol moieties and typically by the PEG-related outward shifting of the slipping/shear plane [28]. The active ingredients DMS and OLE-GS loaded in the nanosystems do not seem to influence the surface charge, pointing to an interaction with the lipophilic internal cavity of the cyclodextrins.

### 2.5. DMS Permeation through Monolayer

**Figure 6:**
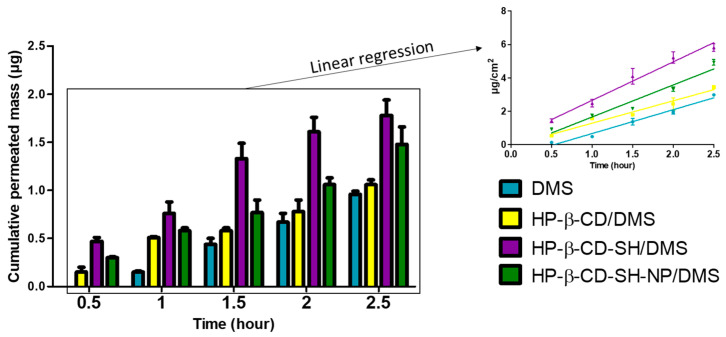
Data on cumulative mass of DMS permeated across ALI cell model (NCI-H441 cell line). Means ± SD of at least 4 runs.

The quantity of DMS deposited on the Transwell^®^ filter after 30 s of nebulization was preliminarily determined, resulting in 5 ± 1 µg for all the formulations tested. Figure 6 shows DMS cumulative permeation through the monolayer for all the formulations under study, and for the control sample, consisting of plain DMS in suspension. The latest allowed the calculation of the parameters reported in Table 3. As can be seen from Figure 6 and from the flux data reported in Table 3, when the DMS is complexed to HP-β-CD-SH, there is an increase in the cumulative mass of permeated drug over time, both compared to the control and to the HP-β-CD/DMS and HP-β-CD-SH-NP/DMS formulations. The formation of disulfide bridges between -SH groups of the cyclodextrin and those of the mucin glycoproteins allow a prolongation of the residence time of the drug at the administration site, with a consequent increase in bioavailability [29]. Physiological mucus presence in lung is often associated with hypermucosis for several inflammatory lung diseases (i.e., asthma, cystic fibrosis, bronchitis, and chronic obstructive pulmonary disease). The NCI-H4441 ALI model is representative of physiological conditions, being cells with typical cuboidal morphology of type II pulmonary epithelial cells, displaying microvilli, and secreting a wide variety of glycoproteins, such as mucins. Among these, mucin 5AC is greatly represented, similarly to what occurs on the ocular surface [30].

At shorter times, DMS permeation has the following trend: HP-β-CD-SH/DMS > HP-β-CD-SH-NP/DMS > HP-β-CD/DMS. However, at longer times, a significant variation is not observed between HP-β-CD-SH/DMS and HP-β-CD-SH-NP/DMS. This is demonstrated by the T2.5 values reported in Table 3, which are not significantly different from each other but significantly greater than those of the control and of HP-β-CD/DMS. The data relating to lag times (L) (Table 3) also show a significantly higher value for HP-β-CD-SH-NP/DMS compared to HP-β-CD-SH/DMS and HP-β-CD/DMS, indicating the need for a longer time to pass before the quasi-steady state is established, i.e., for the cellular monolayer to be saturated with the drug. This difference can be attributed to a greater rigidity of the nanoparticles compared to non-cross-linked cyclodextrins, while it seems that at longer times the biological mechanism by which the nanoparticles are internalized has a greater influence on the DMS permeation process. The data relating to HP-β-CD-SH/DMS are in agreement with those obtained in vivo with the rabbit eye, reported in a previous paper [12]. There, it was hypothesized that the better permeation of DMS complexed to HP-β-CD-SH, compared to that obtained with the non-thiolated cyclodextrin, could depend not only on the mucoadhesion of the cyclodextrin but also on its ability to promote absorption [31]. All these data indicate that, regarding DMS permeation, thiolation alone represents an advantage over the subsequent cross-linking reaction to form HP-β-CD-SH-NP.

### 2.6. Evaluation of the Protective Effect from Oxidative Stress of OLE-GS Nebulized on the Monolayer

The amount of OLE-GS deposited on the Transwell^®^ filter after various nebulization times was determined. The test was carried out at 15, 20, and 30 s of nebulization, obtaining a nebulized quantity of 11.4 ± 2.8, 50.0 ± 7.5, and 142.4 ± 11.7 µg, respectively. The 15 s of nebulization was chosen when performing the subsequent tests, because it guaranteed a non-cytotoxic dose of nebulized OLE-GS.

**Figure 7:**
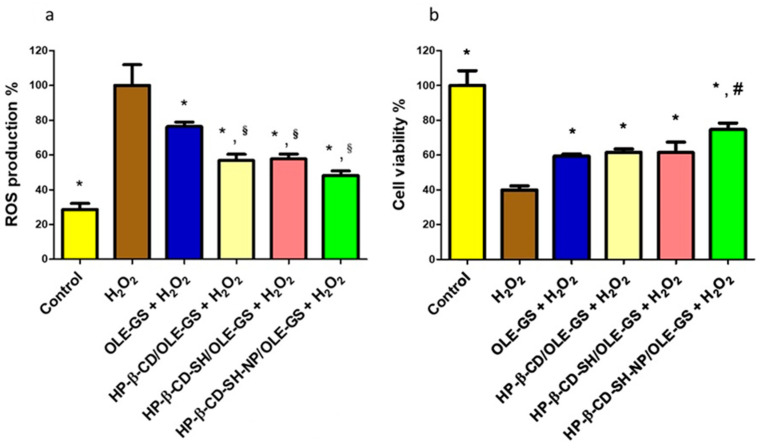
(**a**) Reactive oxygen species (ROS) production in NCI-H441 pre-treated for 2 h with nebulized OLE-GS complexed or not with all types of cyclodextrins in HBSS and subsequently treated with 250 μM H_2_O_2_ for 1 h. Data are expressed as % ROS production compared to 100% (cell treated with H_2_O_2_). (**b**) NCI-H441 viability after 2 h pre-treatment with nebulized OLE-GS complexed or not with all type of cyclodextrins in HBSS, and subsequent 1 h treatment with 250 μM H_2_O_2_. Data are expressed as % viable cells compared to negative control (H_2_O_2_). * *p* < 0.05 from H_2_O_2_; § *p* < 0.05 from OLE-GS + H_2_O_2_; # *p* < 0.05 from all other.

Figure 7a shows data for ROS production in the NCI-H441 cell line-based ALI lung model, subjected to oxidative stress, and pretreated or not with OLE-GS-based samples (either alone or in cyclodextrin complexes). ROS production after cell treatment with H_2_O_2_ is significantly higher than ROS production in cells incubated with control (normal medium). All samples tested managed to significantly reduce the production of ROS. Moreover, when OLE-GS is complexed with cyclodextrins, it limits the production of ROS significantly more effectively than when it is not complexed. No significant differences were found between the various formulations under study, though. In Figure 7b, it is reported that the cell mortality due to the H_2_O_2_ induced oxidative stress. The pre-treatment of the monolayer with the formulations allows for a significant reduction in cell mortality. The effect of cyclodextrin on stabilizing labile compounds in biological environments has been reported; e.g., methyl-β-cyclodextrin has shown the ability to protect the antioxidant molecules contained in bergamot essential oil [32], as well as the peptide dalargin [24]. These effects were evidenced despite the fact that the hydrophobic cavity of the CD could not host the entire molecule, but only a hydrophobic portion of it. This could be the case with OLE-GS, which, in its composition, contains many high molecular weight polyphenols [33]. In particular, HP-β-CD-SH-NP/OLE-GS is significantly more efficient than all the other formulations tested. This finding suggests that the complexation of OLE-GS with HP-β-CD-SH-NP can more stably protect the antioxidant molecules from degradation. This hypothesis is in agreement with what was observed with dalargin complexed with CD-based nanoparticles, compared to CD as the macromolecular carrier [34].

## 3. Materials and Methods

### 3.1. Materials

Hydroxypropyl-β-cyclodextrin (HP-β-CD) MW 1380 Da (SR: 0.6), dexamethasone (DMS), thiourea, Ellman’s reagent (2,2′-dinitro-5,5′-dithiobenzoic acid), l-cysteine hydrochloride monohydrate, tris(hydroxymethyl)aminomethane, Sephadex G-15, RPMI-1640 medium (RPMI), Insulin–Transferrin–Selenium (ITS), trypsin–EDTA and Triton X-100, a mixture of antibiotics consisting of an aqueous solution of penicillin (10,000 U/mL) and streptomycin (10,000 µg/mL), 10 mM phosphate buffer pH 7.3 without Ca^2+^ and Mg^2+^ (PBSA), Hanks’ Balanced Salt Solution (HBSS), Dulbecco phosphate-buffered saline (DPBS), DMSO, DMSO-d6, D_2_O, and a trypsin–EDTA buffer solution containing 0.25% trypsin were all purchased from Sigma-Aldrich (Darmstadt, Germany). The reagents 1,8-Bis-Maleimidotriethylene glycol (BM(PEG)2), PM: 308.29 g/mol and 1,11-Bis-Maleimidotriethylene glycol (BM(PEG)3), PM: 352.34 g/mol were purchased from Thermo Fisher Scientific (Waltham, MA, USA). Cell proliferation reagent (WST-1) was provided by Roche diagnostic (Milan, Italy). Human lung adenocarcinoma NCI-H441 epithelial cell line was purchased from the American Type Culture Collection LGC standards ((ATCC HTB-174), Milan, Italy) and propagated as indicated by the supplier. Olive leaf extracts of the Giarraffa varieties subjected to water stress (OLE-GS) was supplied by Life Sciences Department of the University of Siena, Siena (SI), Italy and previously characterized [33].

### 3.2. Synthesis and Purification of Thiolated Hydroxypropyl-β-Cyclodextrin (HP-β-CD-SH)

The synthesis and purification of HP-β-CD-SH was performed as previously described [12]. Briefly, 400 mg of HP-β-CD were solubilized in 2 mL of 10% acetic acid, and 2.14 g of thiourea were dissolved at 40 °C under stirring in 8 mL of 0.44 M HCl. Subsequently, the thiourea solution was added dropwise to that of HP-β-CD. The resulting mixture was irradiated in a microwave device (Microwave Biotage Initiator, Uppsala, Sweden) with temperature regulation and maximum power set at 90 watts. Irradiation was continued for 5 min at 87 °C and for 50 min at 80 °C. After this first phase, hydrolysis of the thiourea residue was started by adding a few drops of 10 M NaOH until a pH of approximately 8–9 was reached. The resulting mixture was further irradiated for 3 min at 80 °C. The product was collected by solvent evaporation under vacuum (rotary evaporator, BÜCHI, Milano, Italy) at 30–40 °C. Subsequently, the solid obtained was washed five times with 20 mL of acetone, followed by centrifugation for 20 min (5000 rpm; room temperature, IEC MICROCL 17, Termo Fisher Scientific). The sediment was freeze-dried (VirTis freeze-dryer, freezing temperature −40 °C, drying at 30–40 mTorr, up to 16 °C) and subsequently dissolved in Milli-Q water at a concentration of 100 mg/mL. Purification was completed by size exclusion chromatography, using a 30 × 1.5 cm chromatographic column packed with Sephadex G-15 resin (22 cm) and Milli-Q water as the mobile phase. The thiolation degree was assessed by ^1^H-NMR measurements with a Brucker 400 UltraShield™ spectrometer operating at 400 MHz for the ^1^H core. During acquisition, the temperature (18 °C) was controlled via the Varian control unit (accuracy ± 0.1 °C). The HP-β-CD-SH was solubilized in D_2_O at a concentration of 10 mg/mL. The areas assigned to the anomeric protons of thiolated and non-thiolated sugar residues were integrated and compared. The percentage of thiolated sugars was calculated by comparing the areas of thiolated anomeric sugars with the sum of all the anomeric areas [13]. The formation of cyclodextrin aggregates was assessed on HP-β-CD-SH dispersions in DPBS, concentrations ranging from 5 to 20 mg/mL. Aggregate diameter distribution was evaluated by light scattering (Zetasizer nano series Malvern, Malvern, PA, USA), at 25 °C in HBSS solution.

### 3.3. Synthesis and Purification of Nanoparticles Based on HP-β-CD-SH (HP-β-CD-SH-NP)

Either BM(PEG)2 or BM(PEG)3 was used to convert stabilized HP-β-CD-SH clusters into nanoparticles. A 10 mg/mL HP-β-CD-SH solution in HBSS was left overnight under stirring at ambient temperature, after which variable volumes of a 6.2 mg/mL BM(PEG)2 solution or a 7 mg/mL BM(PEG)3 solution, both in DMSO, were added. Following the addition, the stirring was continued for 2hr at 4 °C on a tilting stirrer. The reactions were stopped by adding 50 µL of 62 mg/mL cysteine in HBSS solution for every ml of reaction. The dispersion was incubated at room temperature for 15 min, then ultracentrifuged (Thermo Fisher Sorvall MTX150) at 100,000 rpm for 20 min at 4 °C, thus obtaining a pellet with a gelatinous consistency. For purification, the HP-β-CD-SH-NP pellet dispersed in 1 mL of Milli-Q water was filtered 3 times (Avanti Polar Lipids mini extruder, Sigma-Aldrich, Darmstadt, Germany) through a filter with a diameter of 0.1 μm. After each filtration, approximately 0.8 mL of solvent was discarded, and the volume was brought back to 1 mL with Milli-Q water before repeating the filtration. For the cell viability assays, HP-β-CD-SH-NP was diluted to the desired concentration with RMPI medium. For the nebulization tests, the sample was diluted with HBSS.

### 3.4. Determination of the Association Constant for Cyclodextrins/DMS and Cyclodextrins/OLE-GS

The Benesi–Hildebrand method was used as previously described [13]. Dispersions of HP-β-CD, HP-β-CD-SH, or HP-β-CD-SH-NP in HBSS in the 0–100 mg/mL concentration range were prepared. To determine the cyclodextrin/DMS association constant, 500 µL of a 5.8 µM DMS solution in water was added to 500 µL of each cyclodextrin-based dispersion. These were left to stir overnight at room temperature. The same procedure was repeated to determine the cyclodextrin/OLE-GS association constant by adding 500 µL of 0.6 mg/mL OLE-GS in water to 500 µL of each cyclodextrin-based dispersion. The variation of UV absorbance was monitored at 258 nm and 290 nm for DMS and OLE-GS, respectively. All values were normalized on the ABS value at 600 nm to avoid scattering interference.

### 3.5. Preparation of Drug-Loaded HP-β-CD, HP-β-CD-SH, or HP-β-CD-SH-NP Samples

The loading of DMS or OLE-GS in HP-β-CD-SH-NP was carried out by adding each drug to a preformed cyclodextrin derivative dispersion. HP-β-CD, HP-β-CD-SH, or HP-β-CD-SH-NP were first dispersed in HBSS (10 mg/mL). To one mL of each of these dispersions, 10 μL of 25 mg/mL DMS in ethanol, or 10 μL of 30 mg/mL OLE-GS in water, were added to form suspensions, which were left to incubate overnight at room temperature. At the end of the preparation, the diameter distribution and ζ potential of the dispersed particles were determined by light scattering (Zetasizer nano series Malvern). Three measurements were carried out for each sample, and the data obtained were analyzed in terms of average diameter expressed in percent cumulative intensity and polydispersity index (PDI).

### 3.6. Biological Investigation

In vitro biological evaluations were conducted on human lung adenocarcinoma NCI-H441 epithelial cell line. NCI-H441 cells were grown in RPMI medium with 1% pen/strep and 10% FBS at 37 °C in a 5% CO_2_ atmosphere, as previously described [35].

#### 3.6.1. Cell Viability Assay

Cytotoxicity studies were performed using the WST-1 assay as previously described [17]. Cells were seeded onto 96-well plates, at a concentration of 4 × 10^4^ cells per well, and allowed to proliferate for 24 h at 37 °C, 5% CO_2_. After 24 h, the culture medium was removed and replaced with the samples under study. The effect of DMS on cell viability was evaluated in a concentration range between 0.02 and 2 mg/mL, those of HP-β-CD and HP-β-CD-SH between 5 and 100 mg/mL, and that of OLE-GS in a range between 0.001 and 0.5 mg/mL. After 4 h of incubation, cells were washed with RPMI. Culture medium was then added and incubated at 37 °C for 2 h with WST-1. The amount of formazan produced was evaluated at 450 nm. After the cytotoxicity screening, the effects of DMS or OLE-GS loaded CD-based samples on cell viability were verified for the following selected formulations:

- 0.25 mg/mL DMS containing 10 mg/mL of HP-β-CD (code HP-β-CD/DMS);
- 0.25 mg/mL DMS containing 10 mg/mL of HP-β-CD-SH (code HP-β-CD-SH/DMS);
- 0.25 mg/mL DMS containing 10 mg/mL of HP-β-CD-SH-NP (code HP-β-CD-SH-NP/DMS);
- 0.3 mg/mL OLE-GS containing 10 mg/mL of HP-β-CD (code HP-β-CD/OLE-GS);
- 0.3 mg/mL OLE-GS containing 10 mg/mL of HP-β-CD-SH (code HP-β-CD-SH/OLE-GS);
- 0.3 mg/mL OLE-GS containing 10 mg/mL of HP-β-CD-SH-NP (code HP-β-CD-SH-NP/OLE-GS).

#### 3.6.2. In Vitro Air–Liquid Interface (ALI) Model

For the permeation experiment through the monolayer, the in vitro Air–Liquid Interface (ALI) model was used, as reported by Vizzoni et al. [35]. Briefly, NCI-H441 cells were seeded at a concentration of 2.5 × 10^4^ cells per well onto 12-well Transwell^®^ plates with 0.4 µm pore polyester filters and incubated for 10 days at 37 °C in a 5% CO_2_ atmosphere. One and a half mL of fresh medium was placed in the basolateral chamber, while a 0.5 mL volume containing cells was added to the apical chamber. The medium was changed every 2 days. Forty-eight hours after seeding, the medium in the apical chamber was removed, while that in the basolateral chamber was replaced with a polarizing RPMI medium, containing 1% ITS and 200 nM DMS. Cell monolayer confluence and integrity were monitored by transepithelial electrical resistance (TEER) measurements and verified at the end of every experiment.

#### 3.6.3. Permeation of Nebulized DMS Formulations through ALI Lung Model

HP-β-CD/DMS, HP-β-CD-SH/DMS, HP-β-CD-SH-NP/DMS, and DMS (0.25 mg/mL) in HBSS were directly nebulized on the ALI cell model [35]. The nebulization of the various samples was carried out using an Aerogen Solo^®^ mesh nebulizer (Galway, Ireland). One ml of each sample was loaded in the cup, and nebulization was performed for 30 s.

All experiments were performed in triplicate. At intervals of 0.5 h to 2.5 h, 500 µL was withdrawn from the basolateral chamber and replaced with fresh HBSS. The samples were analyzed by high-performance liquid chromatography as previously described [12]. TEER was measured at time 0 and hourly for 3 h. To quantify the amount of DMS impacting on the ALI cell model, the method already reported by Vizzoni et al. was applied [35]. Briefly, the nebulization test was carried out for 30 s on a filter placed inside Transwell^®^ supports. Subsequently, the filter was placed in HBSS and stirred in a shaker bath for one hour. The filter was then removed, and the supernatant was analyzed spectrophotometrically at 242 nm. The procedure was performed in triplicate.

Permeation data were treated as previously described [36], assuming that the volume of sample nebulized onto the cell monolayer was identical for all samples under study. Briefly, for each permeation through cell monolayer, a value of permeation flux was calculated from the following equation:

Flux = dM/dt 1/A(2)

that is the slope of the linear portion of the cumulative amount permeated per unit surface area (0.33 cm^2^) vs. time plot. For each plot, the linear regression analysis was extended to the set of data points that gave the best fit, as judged from the R^2^ value. This, in all of the cases investigated, was >0.9. The average cumulative amount permeated per unit area was calculated to track each permeation profile and determine T2.5h, that is, the cumulative transport per unit area for the entire duration of the experiment. The lag time (L), that is, the time axis intercept of the linear regression, was also determined for each plot.

#### 3.6.4. Protective Effect of Nebulized OLE-GS Formulations on H_2_O_2-_Stressed ALI Lung Model

The evaluation of the protective effect of OLE-GS from oxidative stress was carried out as already described [37]. HP-β-CD/OLE-GS, HP-β-CD-SH/OLE-GS, HP-β-CD-SH-NP/OLE-GS and OLE-GS (0.3 mg/mL) in HBSS were directly nebulized on the ALI cell model [35]. The nebulization of the various samples was carried out using an Aerogen Solo^®^ mesh nebulizer (Galway, Ireland). One ml of each sample was loaded in the cup and nebulization was performed for 15 s.

After deposition onto the monolayers, ALI monolayers were incubated for 2 h and washed with fresh medium. Then, the oxidative stress was produced by incubation for 1 h with 250 µM H_2_O_2_. Cell viability was evaluated after removing the supernatant, washing with fresh medium, and adding the WST-1 reagent.

To evaluate ROS production, the previously removed supernatant was placed in a 96-well plate, the fluorescent probe 2,7-dichloro-di-hydro-fluorescein diacetate, acetyl ester (CM-H2DCFDA) was added at a concentration of 10 µM/well dissolved in PBS. The plate was kept in the dark at room temperature for 30 min, and the fluorescence was subsequently determined at an excitation wavelength of 488 nm and emission of 510 nm. The amount of nebulized OLE-GS impacting on ALI cell monolayers was determined as described for DMS.

### 3.7. Statistical Analysis

All data are presented as mean ± standard deviation (SD). Three replicates were performed for each experiment concerning the permeation of DMS through the cell monolayer and six for the cell viability experiments. The significance of the difference between two flux, L, or T2.5h values was assessed by the Student’s *t*-test. Three replicates were performed for each experiment concerning the evaluation of the protective effect from nebulized OLE-GS oxidative stress and six for the cell viability experiments. The protective effects of the extracts against oxidative stress damage and ROS production were statistically analyzed with one-way ANOVA, followed by Bonferroni post-test. Significant difference was set for ρ < 0.05. All statistical analyses were performed by using GraphPad Prism (version 10) program (Dotmatics, Bishop’s Stortford, UK).

## 4. Conclusions

Data have demonstrated that HP-β-CD-SH could be exploited for drug delivery to the lung, displaying high cytocompatibility and promotion of drug absorbance through in vitro ALI model, similar to what previously reported for ocular administration [12]. Moreover, a deeper focus on the controlled formation and stabilization of cross-linked HP-β-CD-SH aggregates has confirmed their potential as drug delivery carriers. The stabilized CD nanoaggregates are advantageous over the use of not-stabilized HP-β-CD-SH nanoaggregates when labile molecules (i.e., OLE model) are carried. On the other hand, the processing of HP-β-CD-SH into stabilized nanoaggregates seems not to be crucial when the main limit for drug bioavailability is poor water solubility (i.e., DMS model). In those cases, the application of HP-β-CD-SH is to be preferred and produces effective results.

## Figures and Tables

**Table 1:** Association constants (K_a_) of complexes between HP-β-CD or HP-β-CD-SH or HP-β-CD-SH-NP and DMS or OLE-GS, in aqueous solutions. Means ± SD, *n* = 3.

Drug	HP-β-CD (M^−1^)	HP-β-CD-SH (M^−1^)	HP-β-CD-SH-NP (M^−1^)
DMS	480.1 ± 1.1	1573.9 ± 4.2	639.0 ± 0.5
OLE-GS	233.1 ± 1.2	1418.0 ± 13.9	900.0 ± 6.0

**Table 2:** Diameter distribution (Z average diameter and PDI) and ζ-potential values of HP-β-CD, HP-β-CD-SH, HP-β-CD-SH-NP dispersions, and their corresponding DMS and OLE-GS loaded formulations, in HBSS. Cyclodextrins concentration was always 10 mg/mL, DMS 0.25 mg/mL and OLE-GS 0.3 mg/mL. Mean ± SD, *n* = 3.

Formulation	Z Average (nm)	PDI	ζ-Potential (mV)
HP-β-CD	152.4 ± 5.4	0.408 ± 0.040	−8.05 ± 0.61
HP-β-CD-SH	196.8 ± 3.4	0.275 ± 0.030	−6.89 ± 0.49
HP-β-CD-SH-NP	431.5 ± 7.1	0.443 ± 0.061	−5.33 ± 0.37
HP-β-CD/DMS	200.7 ± 9.4	0.460 ± 0.075	−10.00 ± 0.78
HP-β-CD-SH/DMS	301.4 ± 1.6	0.424 ± 0.064	−6.53 ± 1.14
HP-β-CD-SH-NP/DMS	492.2 ± 5.5	0.461 ± 0.092	−4.51 ± 1.54
HP-β-CD/OLE-GS	165.1 ± 5.1	0.424 ± 0.013	−7.64 ± 0.41
HP-β-CD-SH/OLE-GS	189.3 ± 3.2	0.369 ± 0.029	−9.22 ± 1.25
HP-β-CD-SH-NP/OLE-GS	397.3 ± 6.1	0.473 ± 0.022	−5.66 ± 0.56

**Table 3:** DMS permeation across NCI-H441 cell line ALI lung model. Flux: permeation flux; L: Lag time; T2.5h: cumulative transport over the whole time of experiment (2.5 h). Means ± SD of at least 4 runs.

Formulation	Flux (µg cm^−2^ h^−1^)	L (h)	T2.5h (µg cm^−2^)
DMS	1.43± 0.04	0.53± 0.03	2.91± 0.09
HP-β-CD/DMS	1.33± 0.09	0.04± 0.01	3.21± 0.15
HP-β-CD-SH/DMS	2.30± 0.11 *	−0.04± 0.02 ^a^	5.39± 0.48 ***
HP-β-CD-SH-NP/DMS	1.92± 0.02	0.13± 0.01 **	4.48± 0.55 ***

* *p* < 0.05 from all the other formulations; ** *p* < 0.05 from all the other formulations; *** *p* < 0.05 from DMS and HP-β-CD/DMS. ^a^ L approximately equal to zero indicates that no time needs to pass before the quasi-steady state is established.

## Data Availability

Data is contained within the article or Supplementary Material.

## Supplementary Materials

The following supporting information can be downloaded at: https://www.mdpi.com/article/10.3390/ijms25179394/s1.
