# Supplementary material for: Cross-Linked Thiolated Hydroxypropil-β-Cyclodextrin for Pulmonary Drug Delivery
Source: Int J Mol Sci. 2024 Aug 29;25(17):9394. doi: 10.3390/ijms25179394 (PMC11395519; doi:10.3390/ijms25179394)
Supplement: Supplementary file 1 [file ijms-25-09394-s001.zip › ijms-3144702-supplementary.pdf]

SUPPORTING INFO

Cross-linked thiolated hydroxypropil-β-cyclodextrin for pulmonary drug delivery

Luca Cerri [1,2], Chiara Migone[1], Lucia Vizzoni[1,2], Brunella Grassiri[1], Angela Fabiano[1], Anna Maria Piras[1,3,*], Ylenia Zambito[1,4]

[1]Department of Pharmacy, University of Pisa, Pisa, Italy; [2]Department of Life Sciences, University of Siena, Siena, Italy; [3] Center for Instrument Sharing of the University of Pisa (CISUP), University of Pisa, Italy, [4]Research Centre for Nutraceutical and Healthy Foods "NUTRAFOOD", University of Pisa, Pisa, Italy.

*Correspondence: Anna Maria Piras, Via Bonanno 33, 56126, Pisa, Italy; anna.piras@unipi.it

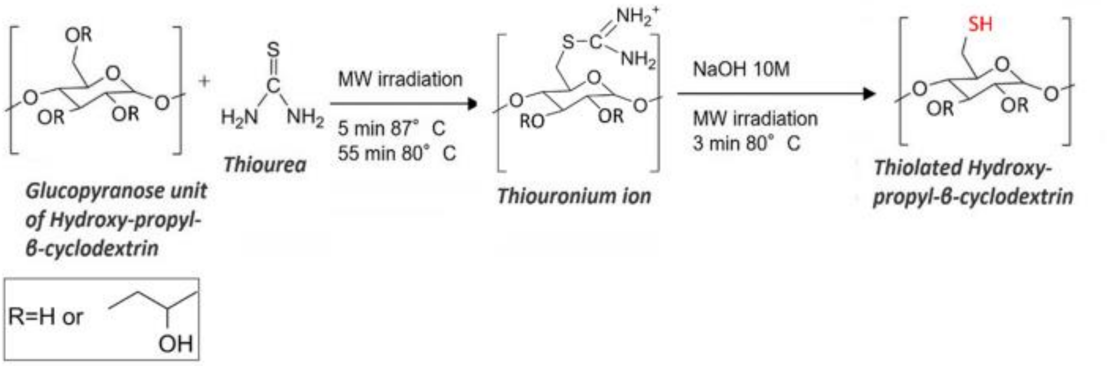

Figure S1. Synthesis scheme for HP-β-CD-SH

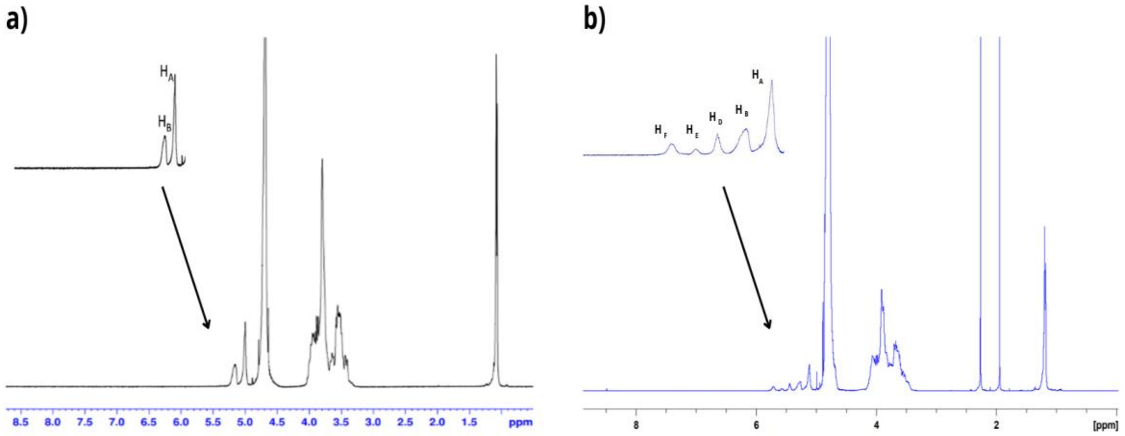

Figure S2. ^{1}H NMR spectra were recorded at 600 MHz in D_2O at 25°C with a concentration of 5 mg/mL. (a) HP-β-CD: the anomeric protons of the hydroxypropyl functionalized and not functionalized glucose at δ = 5.03 ppm and 5.17 ppm, respectively, signals in the range of 2.8-3.9 ppm attributed to the protons of the pyranosidic ring, and at 1 ppm the methylene protons of the hydroxypropyl moiety; (b) HP-β-CD-SH: peaks at δ = 5.03 ppm and 5.169 ppm (corresponding to the anomeric protons of the hydroxypropyl β-CD), along with signals at 5.34 ppm, 5.45 ppm, and 5.61 ppm corresponding to the anomeric signals of thiolated sugars.

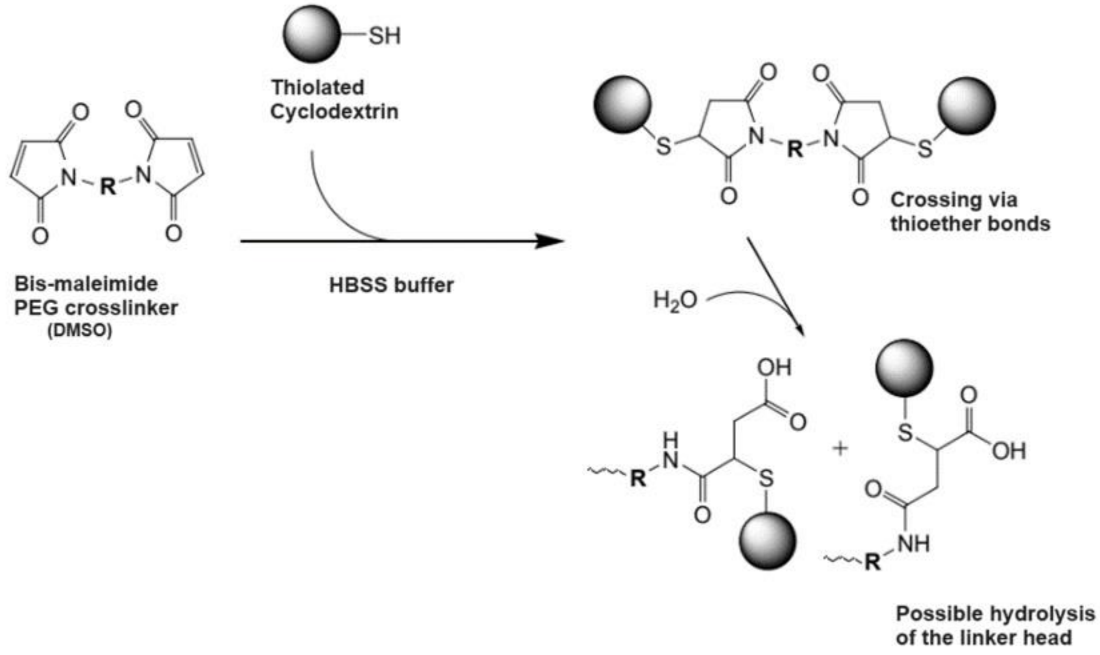

Figure S3. Crosslinking scheme for HP-β-CD-SH-NP.
